# FGFR regulator Memo1 is dispensable for FGF23 expression by osteoblasts during folic acid‐driven kidney injury

**DOI:** 10.14814/phy2.15650

**Published:** 2023-03-26

**Authors:** Katalin Bartos, Matthias B. Moor

**Affiliations:** ^1^ Department of Nephrology and Hypertension Bern University Hospital Bern Switzerland; ^2^ Department of Biomedical Research University of Bern Bern Switzerland; ^3^ National Center of Competence in Research (NCCR) Kidney Control of Homeostasis (Kidney.CH), University of Zurich Zurich Switzerland

**Keywords:** acute kidney injury, FGF23, folic acid, osteoblast, sex difference

## Abstract

Loss of the mediator Of cell motility 1 (*Memo1*) in mice caused kidney disease and a bone disease with diminished osteoblast and osteoclast biomarkers in serum, resembling alterations occurring in adynamic bone disease in humans with chronic kidney disease or in Klotho‐deficient mice. Here, we investigated whether *Memo1* expression in osteoblasts is required for normal bone structure and FGF23 expression. We deleted *Memo1* in the osteoblast–osteocyte lineage in Memo fl/fl mice using a Cre under Col1a1 promotor to obtain osteoblast‐specific knockout (obKO) mice. We studied organs by micro‐computed tomography, qPCR, and western blot. We challenged mice with folic acid for acute kidney injury (AKI) and analyzed organs. Memo obKO were viable without changes in gross anatomy, serum electrolytes, or circulating FGF23 concentrations compared to controls. *Memo1* expression was blunted in bones of Memo obKO, whereas it remained unchanged in other organs. Micro‐CT revealed no differences between genotypes in bone structure of vertebrae, femur, and tibia. During AKI, *Fgf23* expression in calvaria, and renal transcriptional changes were comparable between genotypes. However, renal injury marker expression, circulating FGF23, and parathyroid hormone revealed a sex difference with more severely affected females than males of either genotype. The present data imply that Memo1 in osteoblasts is dispensable for bone structure and expression of *Fgf23*. Moreover, we found evidence of potential sex differences in murine folic acid nephropathy similar to other experimental models of renal injury that are important to consider when using this experimental model of renal injury.

## Graphical abstract

Memo1 in osteoblasts is dispensable for bone structure and renal injury‐driven expression of Fgf23.

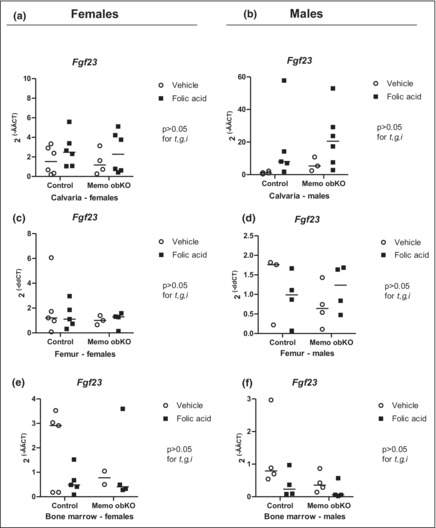

## 1 INTRODUCTION

Mediator of cell motility 1 (Memo1) is an intracellular redox protein with an essential role in several receptor tyrosine kinase signaling pathways and regulating cell migration, redox environment, mineral homeostasis, aging, vascular, and central nervous system development (MacDonald et al., 2014; Marone et al., 2004; Schotanus & Van Otterloo, 2020). *Memo1* is a developmental lethal gene, as *Memo1* homozygous mutants are not viable (Kondo et al., 2014). In a forward‐genetic screen, *Memo1* was found to be required for ossification and vascular invasion of the developing cranial base skeletal elements (Van Otterloo et al., 2016). Postnatal whole‐body deletion of Memo1 caused a phenotype partially resembling *Fgf23*‐ and *Klotho*‐deficient mice with premature aging traits, insulin hypersensitivity, impaired bone structure, and a disordered mineral homeostasis (Haenzi et al., 2014; Kawaguchi et al., 1999; Kuro‐o et al., 1997; Moor, Ramakrishnan, et al., 2018; Shimada et al., 2004). In contrast to Fgf23 and Klotho, Memo1 deletion does not cause hyperphosphatemia and *Memo1* expression is not regulated by minerals or vitamin D (Moor & Bonny, 2020; Moor, Ramakrishnan, et al., 2018). FGF23 is a major regulator of phosphaturia and its gene expression in the bone is upregulated during acute and chronic kidney disease; however, the underlying mechanisms are complex and only partially understood (recently reviewed in Agoro and White, (2023)). We have previously reported that Memo1 in osteoblasts is required for normal ERK signaling in response to FGF2 (Moor, Ramakrishnan, et al., 2018). Kaludjerovic et al. have reported Klotho expression in the bone and a significant role for Klotho in positive feedback‐mediated excessive expression of FGF23 in experimental models of kidney disease (Kaludjerovic et al., 2017). Specifically, Klotho was required to promote excessive FGF23 expression by long bones during kidney disease (Kaludjerovic et al., 2017). Since Memo1 is a regulator of FGFR signaling (Bartos et al., 2020; Haenzi et al., 2014; Moor, Ramakrishnan, et al., 2018), we hypothesized that osteoblast‐specific deletion of Memo1 impairs bone homeostasis and the bone's FGF23 expression in response to renal injury. We have established an osteoblast specific *Memo1* knock‐out mouse model (Memo1 obKO) and found that *Memo1* in osteoblasts is dispensable for both normal bone structure but also FGF23 expression in healthy animals and those challenged with experimental acute renal injury (AKI) using folic acid injection.

## 2 METHODS

### 2.1 Animal studies

Mice were fed a standard laboratory chow (3802 PX Granovit AG, Kaiseraugst, Switzerland; calcium 12 g/kg, phosphorus 8.3 g/kg, sodium 2.3 g/kg, vitamin D 1160 IU/kg, vitamin A 26900 IU/kg, copper 0.4 mg/kg, iron 74 mg/kg, iodine 1.3 mg/kg, protein 24%, crude fat 4.9%, crude fiber 4.7%) and were kept on 12/12 light–dark cycles. All animal experimental protocols were approved by the veterinary service of the Canton of Bern (license number BE47/19).

### 2.2 Generation of osteoblast‐specific Memo1 knock‐out (Memo1 obKO) mice

An osteoblast‐specific Memo1 knock‐out (Memo1 obKO) mouse model was established by crossing mice floxed for exon two of the *Memo1* gene (Haenzi et al., 2014) backcrossed to the C57BL/6J background over at least 10 generations with Col1a1 Cre transgenic mice carrying a Cre recombinase under the control of the 2.3‐kb proximal fragment of the α1(I)‐collagen promoter (Dacquin et al., 2002; Haenzi et al., 2014) obtained through Riken BioResource Research Center (Ibaraki, Japan).

### 2.3 Genotyping

Genotypes were determined by PCR of ear punch biopsy. DNA was extracted with KAPA Express Extrakt (KK7102 Roche, distributed through Merck & Cie, Schaffhausen, Switzerland) following the manufacturer's instructions. PCR reaction was run using AccuStart II PCR SuperMix (95137500 Quantabio, distributed through VWR International AG, Dietikon, Switzerland) and the following primers: Memo1 forward 5′‐TAG GCC ACA GGA TGT CGT TTC‐3′, Memo1 reverse 5′‐GCC GTC TAA AAC TTA CGG TGC‐3′, Cre forward 5′‐ACC AGG TTC GTT CAC TCA TGG‐3′, Cre reverse 5′‐AGG CTA AGT GCC TTC TCT ACA‐3′, ordered from Microsynth. Reactions were run using the following program: 95°C 5 min, 28 cycles (95°C 30 s, 60°C 30 s, 72°C 50 s), 72°C 5 min, and stored at 4°C. Products were visually assessed on 2% agarose gels.

### 2.4 Acute kidney injury (AKI)

Mice, aged 9–10 weeks, were intraperitoneally injected with a single dose of vehicle (300 mM NaHCO_3_) or folic acid (240 mg/kg of body weight) (F7876 Sigma‐Aldrich). Twenty‐four hours later, serum and organs were collected.

### 2.5 Mouse dissection

Memo1 obKO and control mice were dissected at age 9–10 weeks. Folic acid and vehicle injected Memo1 obKO and control mice were dissected 24 h after injection. Mice were anesthetized under isoflurane; the thoracic cage was opened to install a syringe in the left ventricle with a clamp, and blood was collected. Kidneys, liver, heart, and calvaria were collected and snap frozen for RNA and protein extraction. Long bones were collected and either kept in 70% ethanol for micro‐CT analysis and tibias were snap frozen. The epiphyseal ends of femurs were cut off and bone marrow was washed out with a syringe using Hanks' balanced salt solution (HBSS) (H9269 Sigma‐Aldrich). Femur bone shell and bone marrow were snap frozen and all the collected organs were stored at −80°C.

### 2.6 Organ preparation

Tibia and femur bone shells were crushed with a metal pestle in a metal mortar on liquid nitrogen. Tissues were lyzed using Lysing Matrix ceramic beads (116913050‐CF MP Biomedicals, distributed through Lucerna Chem AG) in FastPrep‐24™ Classic Instrument (116004500 MP Biomedicals) for 2× 60 s at 6.5 m/s. Bone marrow samples were spun down at 2000 g for 5 min.

### 2.7 Histology

Kidneys were postfixed in 4% paraformaldehyde, embedded in paraffin, and sections stained with hematoxylin–eosin. Images were acquired on a Nikon Eclipse E600 microscope (Nikon Instruments).

### 2.8 RNA extraction

RNA was extracted using 1 mL of TRIzol reagent (15596026 Invitrogen) according to the manufacturer's protocol. RNA pellets were dried for 10 min and dissolved in 20–100 μL RNAse‐free H_2_O. RNA concentration was measured photometrically using Nanodrop (Nanodrop 2000 Thermo Fisher). Reverse transcription of 500–1000 ng RNA to cDNA was done with PrimeScript RT reaction kit (RR037B Takara Bio Inc.) and the resulting cDNA mix was diluted 5–10 times.

### 2.9 Real‐time quantitative reverse transcription polymerase chain reaction (qPCR)

Quantitative gene transcript analysis was conducted using Fast SYBR Green Master Mix (4385612 Applied Biosystems, distributed through Thermo Fischer) and primers ordered from Microsynth on QuantStudio 1 (Applied Biosystems). Primer sequences are shown in Table 1. Samples were run in triplicates in 20 μL total volume for each gene with the following program settings: 95°C 20 s, 40 cycles (95°C 1 s, 60°C 20 s), and for melting curve stage: 95°C 1 s, 60°C 20 s, rising at 1% ramp speed to 95°C (1 s), 60°C 1 s. Melting curves were obtained for every run. *β‐Actin* gene expression of the samples was used for normalization. Data were analyzed using the delta–delta CT method.

**TABLE 1:** Primers used for qPCR.

Oligonucleotide	5′−3′
Β‐actin forward	GTC CAC CTT CCA GCA GAT GT
Β‐actin reverse	AGT CCG CCT AGA AGC ACT TGC
Cre forward	CGG TCT GGC AGT AAA AAC TAT
Cre reverse	CAG GGT GTT ATA AGC AAT CCC
Cyp24a1 forward	TAC GCT GCT GTC ACG GAG C
Cyp24a1 reverse	TCT GGA TTT CCC GGA GAA GTC
Cyp27b1 forward	CCT CTG CCG AGA CTG GGA
Cyp27b1 reverse	TCC CGA AAA AGG AAG TGG GT
Fgf23 forward	TAT GGA TCT CCA CGG CAA C
Fgf23 reverse	GTC CAC TGG CGG AAC TTG
Kim‐1 forward	TCC ACA CAT GTA CCA ACA TCA A
Kim‐1 reverse	GTC ACA GTG CCA TTC CAG TC
Klotho forward	CAG CTC CAG GCT CGG GTA
Klotho reverse	AGG TGT TGT AGA GAT GCC AGA CTT
Memo1 forward	AGG ACC TCA GCT GAA CGC T
Memo1 reverse	GGC TCT AGC AGG TCT TTT CG

### 2.10 Protein isolation

Organs were lyzed in 250–500 μL Ripa buffer (R0278 Sigma‐Aldrich) containing protease inhibitors (cOmplete Mini Protease Inhibitor Cocktail 11836153001 Roche), using Lysing Matrix ceramic beads, and cells were lyzed in 200 μL Ripa buffer containing protease inhibitors by 1 h incubation on a shaker, followed by 10,000 g 10 min centrifugation at 4°C and supernatants were stored at −80°C. Protein concentrations were measured with Pierce BCA Protein Assay Kit (23227 Thermo Fisher) on a spectrophotometer at 540 nm (Sunrise, Tecan Trading AG, Männedorf, Switzerland), following the manufacturer's protocol.

### 2.11 Western blot

Fifty microgram protein in Ripa buffer and sample buffer from 2× stock (62.5 mM Tris–HCl pH 6.8, 2% SDS, 25% glycerol, 0.01% bromophenol blue, 10% β‐mercaptoethanol) was denatured for 5 min at 95°C. Samples were loaded on 10% or 12% SDS–PAGE gels and migrated at 80 V for 15 min, then 120 V and transferred on nitrocellulose (88018 Thermo Fisher) at 15 V for 45 min. Proteins were stained by Ponceau staining. Membranes were washed in TRIS‐buffer saline containing 0.1% Tween (TBST) (100 mM Tris base, 3 M NaCl, 2% Tween 20) and blocked for 30 min in 5% non‐fat dried milk in TBST, except membranes probed with phospho‐specific primary antibodies which were blocked in 5% bovine serum albumin (A9647 Sigma‐Aldrich) in TBST. Membranes were incubated overnight at 4°C with monoclonal mouse anti‐Memo1 antibody (Haenzi et al., 2014) kindly provided by Prof. Olivier Bonny, University of Lausanne, washed with TBST, and incubated with 1:10,000 Peroxidase AffiniPure Donkey Anti‐Mouse IgG (H+L) (715035‐150 Jackson ImmunoResearch) for 1 h at room temperature and washed with TBST. Signals were detected using Pierce ECL Western Blotting Substrate (32106 Thermo Fisher) and developed on ChemiDoc XRS+ System (Bio‐Rad Laboratories, Inc.). Bands were quantified using ImageJ.

### 2.12 Biochemical analyses

An alkaline phosphatase assay kit (Colorimetric) (ab83369 Abcam) kit was used for mouse sera according to manufacturer's instructions. Serum electrolytes and urea were measured at the Central Chemistry Laboratories of University Hospital Bern. The following ELISA kits were used according to the manufacturer's protocol: Mouse/Rat FGF‐23 (C‐Term) (606300 Quidel); Mouse/Rat FGF‐23 (Intact) (606800 Quidel); Mouse PTH 184 (602305 Quidel).

### 2.13 Micro‐computed tomography (μCT)

For ex vivo μCT, femora were scanned on a Scanco μCT40 machine (SCANCO Medical) in 70% ethanol with voxel size 6 μm, filter AI 12.3 mm, exposure 300 ms, voltage 70 kVp and current 114 μA. Femur sections 0.5–1.5 mm from distal growth plate for metaphyseal bone and 0.32 mm mid‐shaft (calculated using distal growth plate and minor trochanter as landmarks) underwent automated segmentation into cancellous and cortical bone with grayscale thresholds of 180–1000 and 200–1000. Trabecular bone of vertebral body L4 and L5 were assessed in automatically interpolated regions of interest between three manually selected elliptic planes confined to the trabecular area. Images were analyzed by the manufacturer's software Direct (No model) mode which calculates using distance transformation without assuming anything about the shape of the bone (2D) and TRI (Plate model) mode which is based on triangularization of surface (3D) that assumes that the bone is a stack of parallel plates of material. Analyzed parameters are given according to standard nomenclature (Bouxsein et al., 2010).

### 2.14 Statistical analyses

Analyses of two groups variables were conducted by *t*‐test or Kruskal–Wallis test wherever appropriate. Analyses of two‐factorial experimental designs (four groups) were made using two‐way ANOVA. Analyses were performed in GraphPad Prism 9.3.1. Two‐tailed *p*‐values of <0.05 were considered significant.

## 3 RESULTS

### 3.1 Establishing and validating the specificity and efficacy of an Memo1 obKO mouse model

**FIGURE 1:**
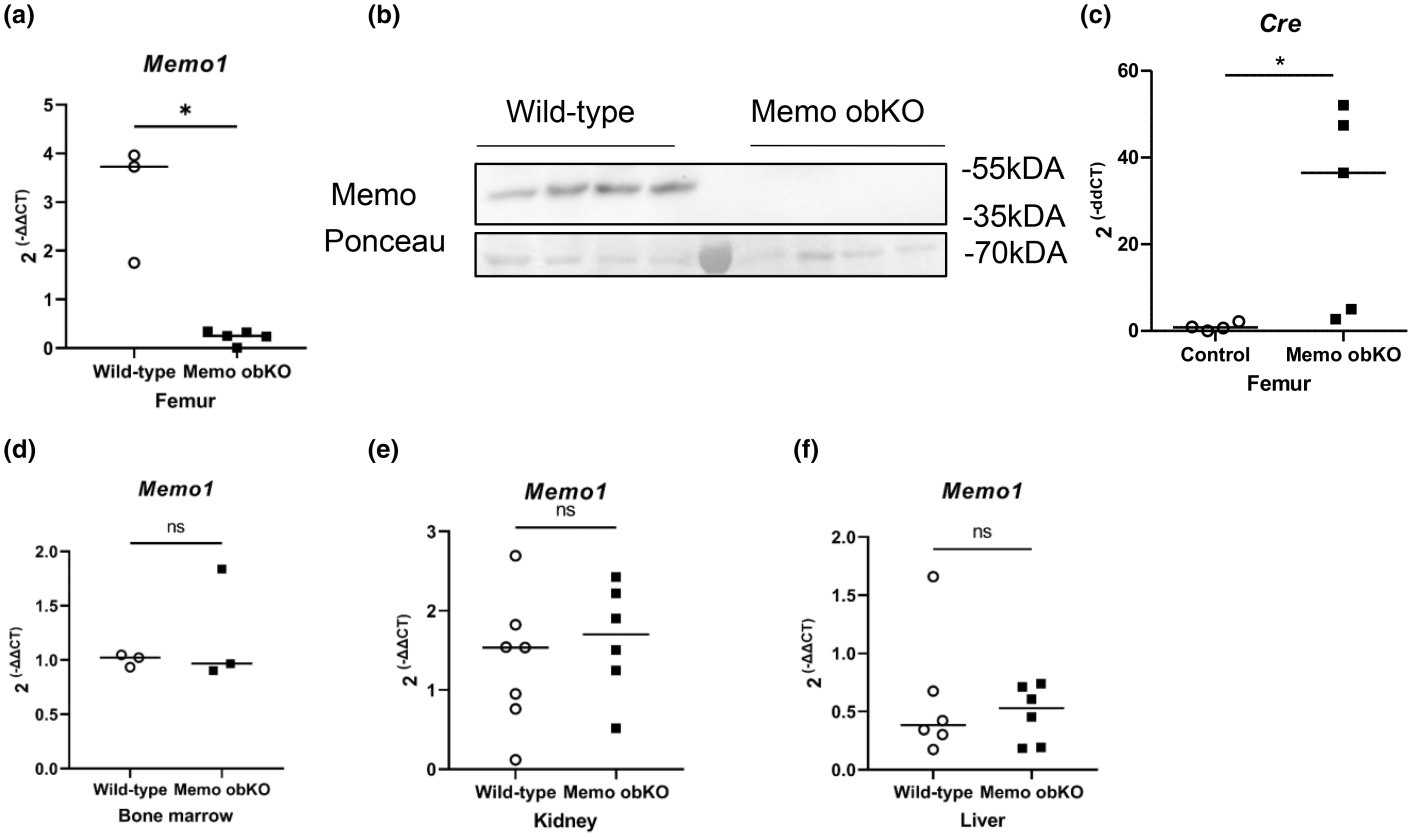
Validation of Memo obKO mouse model. Femoral *Memo1* gene expression (a) and protein abundance (b) were blunted in Memo obKO, whereas Cre expression was detectable (c) in marrow‐free femur of Memo obKO. *Memo1* expression was conserved in bone marrow (d), kidney (e) and liver (f) of Memo obKO animals relative to controls. Statistical analyses by Mann–Whitney *U* test. *, *p* < 0.05; ns, not significant. N of animals is indicated by the number of scatters. Animals were aged 9 weeks and both sexes at equal ratios in each group.

We created an osteoblast‐specific Memo1 knock‐out model (Memo1 obKO) that requires mice carrying loxP sites flanking *Memo1* exon 2 and a Cre recombinase under the control of the α1(I)‐collagen promoter (Dacquin et al., 2002; Haenzi et al., 2014). Memo1 obKO and Cre‐negative littermate control mice were genotyped by tail genomic DNA after dissection. We verified that Memo1 obKO lost *Memo1* gene expression in the bones only and that control mice still express *Memo1* in their bones. To do that, *Memo1* gene expression was measured in bone marrow‐free femur, bone marrow, kidney, and liver by qPCR and Memo1 protein levels of marrow‐free femur were measured by Western blot. *Memo1* expression was blunted in the femur of Memo1 obKO (Figure 1a). The presence of Memo1 protein was not found in Memo1 obKO femur, while it remained detectable in control animals (Figure 1b). Conversely, *Cre* expression was detectable in femur of Memo1 obKO but not of controls (Figure 1c). Memo1 expression remained comparable to controls in bone marrow (Figure 1d), kidney (Figure 1e), and liver (Figure 1f) tissues, signifying Cre non‐expressing tissues remained unaffected. Memo1 obKO were viable without major changes in gross anatomy and development (Figure S1).

### 3.2 Bone structural analysis does not show differences between Memo1 obKO and control mice

**FIGURE 2:**
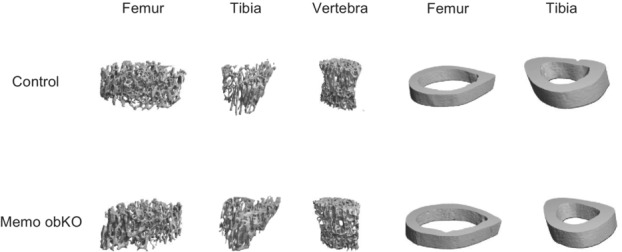
Three‐dimensional reconstructions of bone sites investigated by micro‐CT. Structure was not affected by loss of Memo1 in osteoblasts at any site, and 1 representative image per anatomical site and genotype is shown.

To investigate if Memo1 directly affects the function of osteoblast during bone development and influences bone structure, femur, tibia, and vertebra of Memo obKO and control mice were scanned ex vivo by micro‐computed tomography (μCT). No significant differences between genotypes were present in sex‐disaggregated analyses of trabecular parameters of femoral bone (Tables 2 and 3), tibiae (Tables 4 and 5) and vertebrae L4 and L5 (Tables 6 and 7). Similarly, analyses of mid‐shaft cortical bone of femora (Tables 2 and 3) and tibiae (Tables 4 and 5) revealed no differences between genotypes. A representative 3D reconstruction of each analyzed site is shown in Figure 2.

**TABLE 2:** Femoral μCT analysis of Memo1 obKO males (*n* = 4).

Males			
Femur micro‐computed tomography	Control	Memo1 obKO	*t*‐test
	Mean ± SD	Mean ± SD	
Distal metaphysis trabecular analyses			
Direct (No model)			
BV/TV [1]	0.12 ± 0.03	0.13 ± 0.04	0.24
Conn. D. [1/mm^3^]	313.97 ± 89.65	357.87 ± 79.19	0.29
SMI [1]	2.08 ± 0.38	1.92 ± 0.29	0.25
Tb.N [1/mm]	5.23 ± 0.59	5.53 ± 0.31	0.18
Tb.Th [mm]	0.03 ± 0.00	0.03 ± 0.01	0.68
Tb.Sp [mm]	0.18 ± 03	0.17 ± 0.01	0.17
TRI (Plate model)			
BV/TV [1]	0.12 ± 0.03	0.13 ± 0.04	0.24
BS/BV [1/mm]	78.80 ± 8.87	76.72 ± 11.56	0.48
Tb.N [1/mm]	4.79 ± 0.99	5.17 ± 0.48	0.18
Tb.Th [mm]	0.03 ± 0.00	0.03 ± 0.01	0.47
Tb.Sp [mm]	0.18 ± 0.07	0.17 ± 0.02	0.24
Midshaft cortical analyses			
TRI (Plate model)			
C.Th [mm]	0.17 ± 0.01	0.16 ± 0.01	0.47

Abbreviations: BS/BV, bone surface per bone volume; BV/TV, bone volume per total volume; C.Th, cortical thickness; Conn.D, connectivity density, normed by TV; SD, standard deviation; SMI, structure model index; Tb.N, trabecular number; Tb.Sp, trabecular separation; Tb.Th, trabecular thickness.

**TABLE 3:** Femoral μCT analysis of Memo1 obKO females (*n* = 4).

Females			
Femur micro‐computed tomography	Control	Memo1 obKO	*t*‐test
	Mean ± SD	Mean ± SD	
Distal metaphysis trabecular analyses			
Direct (No model)			
BV/TV [1]	0.06 ± 0.02	0.06 ± 0.03	0.71
Conn. D. [1/mm^3^]	154.42 ± 40.39	169.09 ± 74.93	0.39
SMI [1]	2.60 ± 0.24	2.51 ± 0.33	0.59
Tb.N [1/mm]	4.22 ± 0.25	4.22 ± 0.64	0.74
Tb.Th [mm]	0.03 ± 0.00	0.03 ± 0.00	0.74
Tb.Sp [mm]	0.23 ± 0.01	0.23 ± 0.03	0.81
TRI (Plate model)			
BV/TV [1]	0.06 ± 0.02	0.06 ± 0.03	0.72
BS/BV [1/mm]	90.81 ± 7.98	92.69 ± 12.35	0.86
Tb.N [1/mm]	2.68 ± 0.56	2.82 ± 0.96	0.64
Tb.Th [mm]	0.02 ± 0.00	0.02 ± 0.00	0.94
Tb.Sp [mm]	0.36 ± 0.07	0.34 ± 0.10	0.77
Midshaft cortical analyses			
TRI (Plate model)			
C.Th [mm]	0.15 ± 0.00	0.16 ± 0.00	0.35

Abbreviations: BS/BV, bone surface per bone volume; BV/TV, bone volume per total volume; C.Th, cortical thickness; Conn.D, connectivity density, normed by TV; SD, standard deviation; SMI, structure model index; Tb.N, trabecular number; Tb.Sp, trabecular separation; Tb.Th, trabecular thickness.

**TABLE 4:** Tibia μCT analysis of Memo1 obKO males (*n* = 4).

Males			
Tibia micro‐computed tomography	Control	Memo1 obKO	*t*‐test
	Mean ± SD	Mean ± SD	
Proximal metaphysis trabecular analyses			
Direct (No model)			
BV/TV [1]	0.07 ± 0.01	0.09±0.02	0.44
Conn. D. [1/mm^3^]	99.66 ± 21.31	144.53 ± 64.01	0.37
SMI [1]	2.80 ± 0.17	2.57 ± 0.41	0.74
Tb.N [1/mm]	4.59 ± 0.27	4.91 ± 1.00	0.94
Tb.Th [mm]	0.03 ± 0.00	0.04 ± 0.00	0.62
Tb.Sp [mm]	0.21 ± 0.01	0.19 ± 0.07	0.84
TRI (Plate model)			
BV/TV [1]	0.07 ± 0.01	0.09 ± 0.02	0.44
BS/BV [1/mm]	83.84 ± 2.76	80.44 ± 3.69	0.18
Tb.N [1/mm]	2.84 ± 0.40	3.51 ± 1.00	0.56
Tb.Th [mm]	0.02 ± 0.00	0.02 ± 0.00	0.18
Tb.Sp [mm]	0.33 ± 0.05	0.26 ± 0.14	0.92
Midshaft cortical analyses			
TRI (Plate model)			
C.Th [mm]	0.19 ± 0.01	0.18 ± 0.01	0.80

Abbreviations: BS/BV, bone surface per bone volume; BV/TV, bone volume per total volume; C.Th, cortical thickness; Conn.D., connectivity density, normed by TV; SD, standard deviation; SMI, structure model index; Tb.N, trabecular number; Tb.Sp, trabecular separation; Tb.Th, trabecular thickness.

**TABLE 5:** Tibia μCT analysis of Memo1 obKO females (*n* = 4).

Females			
Tibia micro‐computed tomography	Control	Memo1 obKO	*t*‐test
	Mean ± SD	Mean ± SD	
Proximal metaphysis trabecular analyses			
Direct (No model)			
BV/TV [1]	0.03 ± 0.02	0.04 ± 0.01	0.86
Conn. D. [1/mm^3^]	39.80 ± 31.98	66.55 ± 27.59	0.51
SMI [1]	3.01 ± 0.34	2.87 ± 0.23	0.67
Tb.N [1/mm]	3.51 ± 0.40	3.61 ± 0.43	1.00
Tb.Th [mm]	0.03 ± 0.00	0.03 ± 0.00	0.82
Tb.Sp [mm]	0.28 ± 0.03	0.27 ± 0.04	0.95
TRI (Plate model)			
BV/TV [1]	0.03 ± 0.02	0.04 ± 0.01	0.86
BS/BV [1/mm]	97.86 ± 6.53	100.70 ± 11.16	0.68
Tb.N [1/mm]	1.48 ± 0.64	1.83 ± 0.51	0.80
Tb.Th [mm]	0.02 ± 0.00	0.02 ± 0.00	0.75
Tb.Sp [mm]	0.66 ± 0.21	0.54 ± 0.19	0.71
Midshaft cortical analyses			
TRI (Plate model)			
C.Th [mm]	0.17 ± 0.01	0.18 ± 0.01	0.87

Abbreviations: BS/BV, bone surface per bone volume; BV/TV, bone volume per total volume; C.Th, cortical thickness; Conn.D., connectivity density, normed by TV; SD, standard deviation; SMI, structure model index; Tb.N, trabecular number; Tb.Sp, trabecular separation; Tb.Th, trabecular thickness.

**TABLE 6:** Vertebral μCT analysis of Memo1 obKO males (*n* = 4).

Males			
Vertebral micro‐computed tomography	Control	Memo1 obKO	*t*‐test
	Mean ± SD	Mean ± SD	
L4 vertebra trabecular analyses			
Direct (No model)			
BV/TV [1]	0.19 ± 0.02	0.20 ± 0.02	0.82
Conn. D. [1/mm^3^]	367.30 ± 64.25	360.79 ± 90.91	0.49
SMI [1]	0.57 ± 0.12	0.59 ± 0.14	0.70
Tb.N [1/mm]	5.25 ± 0.41	5.46 ± 0.42	0.62
Tb.Th [mm]	0.04 ± 0.00	0.04 ± 0.00	0.92
Tb.Sp [mm]	0.18 ± 0.02	0.17 ± 0.01	0.50
TRI (Plate model)			
BV/TV [1]	0.20 ± 0.02	0.20 ± 0.02	0.82
BS/BV [1/mm]	64.63 ± 3.37	64.29 ± 5.27	1.00
Tb.N [1/mm]	6.47 ± 0.49	6.17 ± 3.21	0.41
Tb.Th [mm]	0.03 ± 0.00	0.03 ± 0.00	0.96
Tb.Sp [mm]	0.13 ± 0.01	0.12 ± 0.01	0.77
L5 vertebra trabecular analysis			
Direct (No model)			
BV/TV [1]	0.26 ± 0.02	0.24 ± 0.03	0.76
Conn. D. [1/mm^3^]	701.29 ± 63.82	670.33 ± 76.07	0.66
SMI [1]	0.17 ± 0.18	0.29 ± 0.29	0.82
Tb.N [1/mm]	7.02 ± 0.39	6.93 ± 0.61	0.45
Tb.Th [mm]	0.04 ± 0.00	0.04 ± 0.00	0.88
Tb.Sp [mm]	0.13 ± 0.01	0.14 ± 0.02	0.38
TRI (Plate model)			
BV/TV [1]	0.27 ± 0.02	0.24 ± 0.03	0.75
BS/BV [1/mm]	62.19 ± 6.61	63.18 ± 5.83	0.96
Tb.N [1/mm]	8.25 ± 0.36	8.09 ± 0.43	0.50
Tb.Th [mm]	0.03 ± 0.00	0.03 ± 0.00	0.96
Tb.Sp [mm]	0.09 ± 0.00	0.09 ± 0.01	0.53

Abbreviations: BS/BV, bone surface per bone volume; BV/TV, bone volume per total volume; Conn.D., connectivity density, normed by TV; SD, standard deviation; SMI, structure model index; Tb.N, trabecular number; Tb.Sp, trabecular separation; Tb.Th, trabecular thickness.

**TABLE 7:** Vertebral μCT analysis of Memo1 obKO females (*n* = 4).

Females			
Vertebral micro‐computed tomography	Control	Memo1 obKO	*t*‐test
	Mean ± SD	Mean ± SD	
L4 vertebra trabecular analyses			
Direct (No model)			
BV/TV [1]	0.15 ± 0.04	0.16 ± 0.01	0.84
Conn. D. [1/mm^3^]	336.08 ± 143.91	296.34 ± 12.09	0.35
SMI [1]	0.83 ± 0.24	0.81 ± 0.08	0.81
Tb.N [1/mm]	4.52 ± 0.64	4.64 ± 0.15	0.78
Tb.Th [mm]	0.03 ± 0.00	0.04 ± 0.00	0.77
Tb.Sp [mm]	0.21 ± 0.03	0.21 ± 0.01	0.82
TRI (Plate model)			
BV/TV [1]	0.16 ± 0.04	0.16 ± 0.01	0.84
BS/BV [1/mm]	68.93 ± 5.55	67.53 ± 2.50	0.81
Tb.N [1/mm]	5.36 ± 1.02	5.48 ± 0.27	0.79
Tb.Th [mm]	0.03 ± 0.00	0.03 ± 0.00	0.86
Tb.Sp [mm]	0.16 ± 0.03	0.15 ± 0.01	0.97
L5 vertebra trabecular analysis			
Direct (No model)			
BV/TV [1]	0.20 ± 0.01	0.20 ± 0.02	0.95
Conn. D. [1/mm^3^]	579.28 ± 258.67	588.68 ± 177.10	0.96
SMI [1]	0.48 ± 0.08	0.56 ± 0.11	0.22
Tb.N [1/mm]	5.57 ± 0.93	5.55 ± 0.69	0.98
Tb.Th [mm]	0.03 ± 0.00	0.04 ± 0.00	0.90
Tb.Sp [mm]	0.17 ± 0.04	0.18 ± 0.02	0.87
TRI (Plate model)			
BV/TV [1]	0.20 ± 0.01	0.20 ± 0.02	0.94
BS/BV [1/mm]	67.22 ± 5.16	67.01 ± 2.35	1.00
Tb.N [1/mm]	6.67 ± 0.90	6.84 ± 0.74	0.95
Tb.Th [mm]	0.03 ± 0.00	0.03 ± 0.00	0.95
Tb.Sp [mm]	0.12 ± 0.02	0.12 ± 0.02	0.97

Abbreviations: BS/BV, bone surface per bone volume; BV/TV, bone volume per total volume; Conn.D., connectivity density, normed by TV; SD, standard deviation; SMI, structure model index; Tb.N, trabecular number; Tb.Sp, trabecular separation; Tb.Th, trabecular thickness.

### 3.3 Serum electrolytes and mineralotropic hormones unchanged after Memo1 deletion in osteoblasts

We next measured circulating intact and C‐terminal FGF23 in serum by ELISA. This revealed no changes between genotypes (Table 1). Similarly, circulating PTH and serum electrolyte concentrations were comparable between genotypes (Table 8). Further, the renal retention parameter urea remained unchanged in serum (Table 8).

**TABLE 8:** Serum parameters of Memo1 obKO (*n* = 4 per genotype and sex).

	Males		
	Control	Memo1 obKO	*t*‐test
	Mean ± SD	Mean ± SD	
Sodium (mmol/L)	144.00 ± 2.52	140.00 ± 8.43	0.71
Potassium (mmol/L)	5.70 ± 0.96	5.20 ± 0.71	0.26
Calcium (mmol/L)	2.30 ± 0.13	2.14 ± 0.20	0.66
Phosphate (mmol/L)	2.16 ± 0.25	2.01 ± 0.43	0.73
Magnesium (mmol/L)	0.95 ± 0.22	0.86 ± 0.12	0.55
Urea (mmol/L)	8.30 ± 11.76	8.20 ± 1.14	0.57
C‐terminal FGF23 (pg/mL)	200.04 ± 26.02	182.00 ± 46.09	0.62
Intact FGF23 (pg/mL)	293.60 ± 173.48	312.35 ± 100.91	0.78
PTH (pg/mL)	128.87 ± 36.64	158.38 ± 738.24	0.44

	Females		
	Wild‐type	Memo1 obKO	*t‐*test
	Mean ± SD	Mean ± SD	
Sodium (mmol/L)	138.00 ± 16.70	134.00 ± 16.29	0.64
Potassium (mmol/L)	5.20 ± 0.66	5.70 ± 0.70	0.35
Calcium (mmol/L)	1.84 ± 0.42	1.86 ± 0.23	0.83
Phosphate (mmol/L)	1.80 ± 0.41	2.10 ± 0.10	0.17
Magnesium (mmol/L)	0.84 ± 0.17	0.86 ± 0.11	0.65
Urea (mmol/L)	6.90 ± 1.38	7.20 ± 1.15	0.55
C‐terminal FGF23 (pg/mL)	186.58 ± 33.58	173.37 ± 412.03	0.36
Intact FGF23 (pg/mL)	321.18 ± 89.92	291.94 ± 115.89	0.45
PTH (pg/mL)	94.11 ± 61.51	153.62 ± 164.51	0.35

Abbreviation: SD, standard deviation.

### 3.4 Folic acid AKI in Memo1 obKO and control animals

**FIGURE 3:**
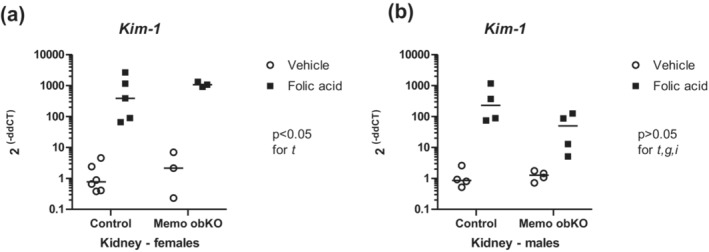
Intraperitoneal folic acid injection induced gene expression of renal injury after 24 h. Kidney injury molecule *Kim‐1* showed increased renal gene expression in folic acid‐treated animals, females (a) more strongly than males (b) of either genotype. Analysis by Two‐ANOVA within each sex. t, treatment effect (folic acid versus vehicle); g, genotype effect; i, interaction between treatment and genotype. N of animals is indicated by the number of scatters. Age of mice was 9 weeks. Data of both sexes pooled are shown in Supplemental Figure 2.

**FIGURE 4:**
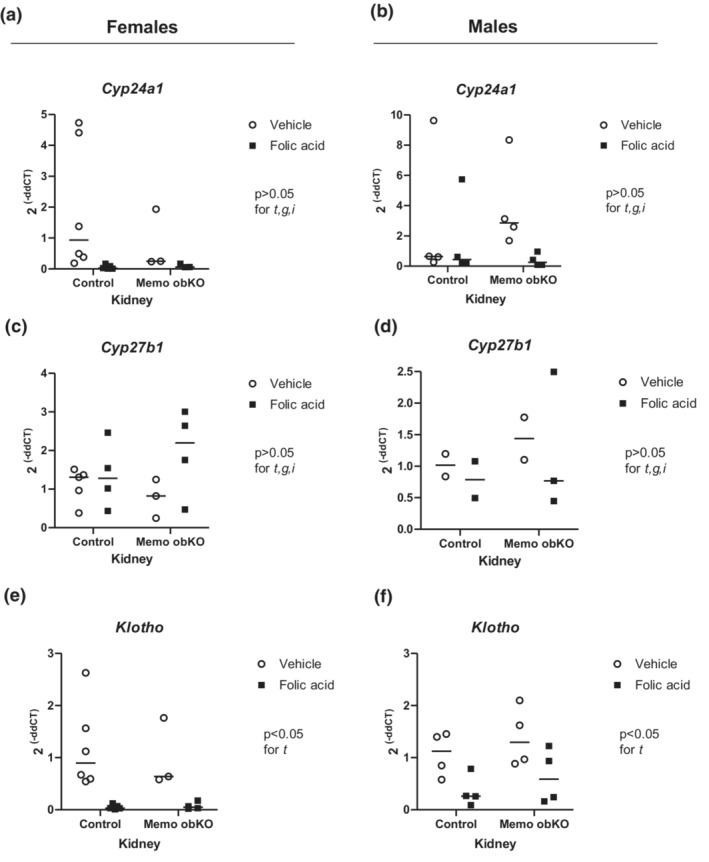
Renal gene expression patterns in AKI. The renal transcripts *Cyp24a1* and *Cyp27b1* encoding 1,25‐(OH)_2_ vitamin D and 25OH‐vitamin D metabolizing enzymes and were assessed by qPCR in female (left panels) and male (right panels) Memo obKO and control mice undergoing folic acid or vehicle injection (a–d). Renal *Klotho* expression was diminished after folic acid treatment in mice of either sex or genotype (e and f). Statistical analyses by Two‐ANOVA within each sex. t, treatment effect (folic acid versus vehicle); g, genotype effect; i, interaction between treatment and genotype. N of animals is indicated by the number of scatters. Age of mice was 9 weeks. Data of both sexes pooled are shown in Figure S3.

Bone‐intrinsic autocrine or paracrine signaling of FGF23 via FGFR/Klotho was reported to be important for the FGF23 increase in experimental kidney disease (Kaludjerovic et al., 2017). To assess a potential role of *Memo1* in kidney disease‐driven Fgf23 expression, we therefore challenged mice of both genotypes with AKI induced by intraperitoneal injection of 240 mg/kg of body weight folic acid. Kidney injury was assessed by k*idney injury molecule‐1* (*Kim‐1*) gene expression (Figure S2). *Kim‐1* was significantly increased in folic acid‐injected Memo1 obKO and control animals of female sex (Figure 3a), but this effect was blunted in males (Figure 3b). We next investigated renal transcripts affected by folic acid AKI as in Egli‐Spichtig et al. (Egli‐spichtig et al., 2018) who reported suppressed renal *Klotho* and *Cyp24a1* expression and increased *Cyp27b1* expression in this model. In our animals, folic acid injection caused a significant decrease in renal gene expression of *Cyp24a1*: In control genotype, folic acid injection caused a 4.5‐fold lower *Cyp24a1* gene expression compared to vehicle, whereas in Memo obKO animals the effect was 9‐fold lower *Cyp24a1* expression (*p* < 0.05 for treatment effect when both sexes are combined, shown in Figure S3a). The sex‐disaggregated data for Cyp24a1 are shown in Figure 4a,b. Renal *Cyp27b1* expression was comparable between Memo1 obKO and control animals, both in combined and sex‐disaggregated data (Figure 4c,d, Figure S3b). Renal *Klotho* expression showed a more than 12‐fold decrease by AKI in control genotype and a 5‐fold decrease in Memo1 obKOs with a *p* < 0.05 for treatment effect in both the combined and the sex‐disaggregated data (Figure 4e,f, Figure S3C).

### 3.5 AKI‐driven FGF23 expression and serum concentrations influenced by sex, but not by genotype

**FIGURE 5:**
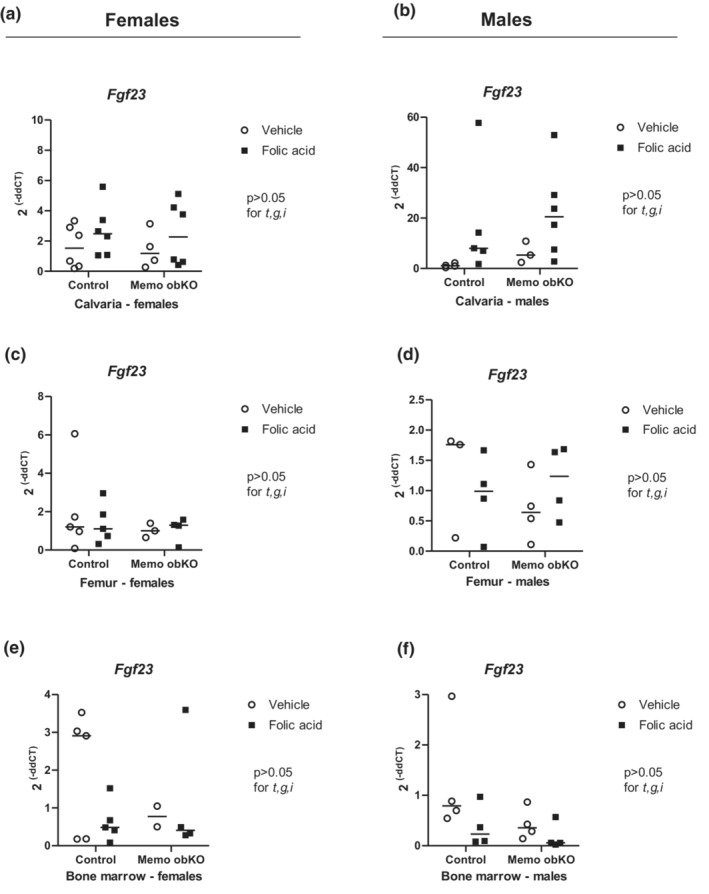
Fgf23 gene expression increased in calvaria after folic acid treatment. Gene expression of *Fgf23* in calvaria (a and b), marrow‐free femur (c and d) and bone marrow (e and f) was assessed by qPCR. Statistical analyses by Two‐ANOVA within each sex. t, treatment effect (folic acid versus vehicle); g, genotype effect; i, interaction between treatment and genotype. Sex‐disaggregated analyses remained at *p* > 0.05, but analysis of both samples combined revealed a treatment effect at *p* < 0.05 for data of panels A and B. N of animals is indicated by the number of scatters. Age of mice was 9 weeks. Data of both sexes pooled are shown in Figure S4.

**FIGURE 6:**
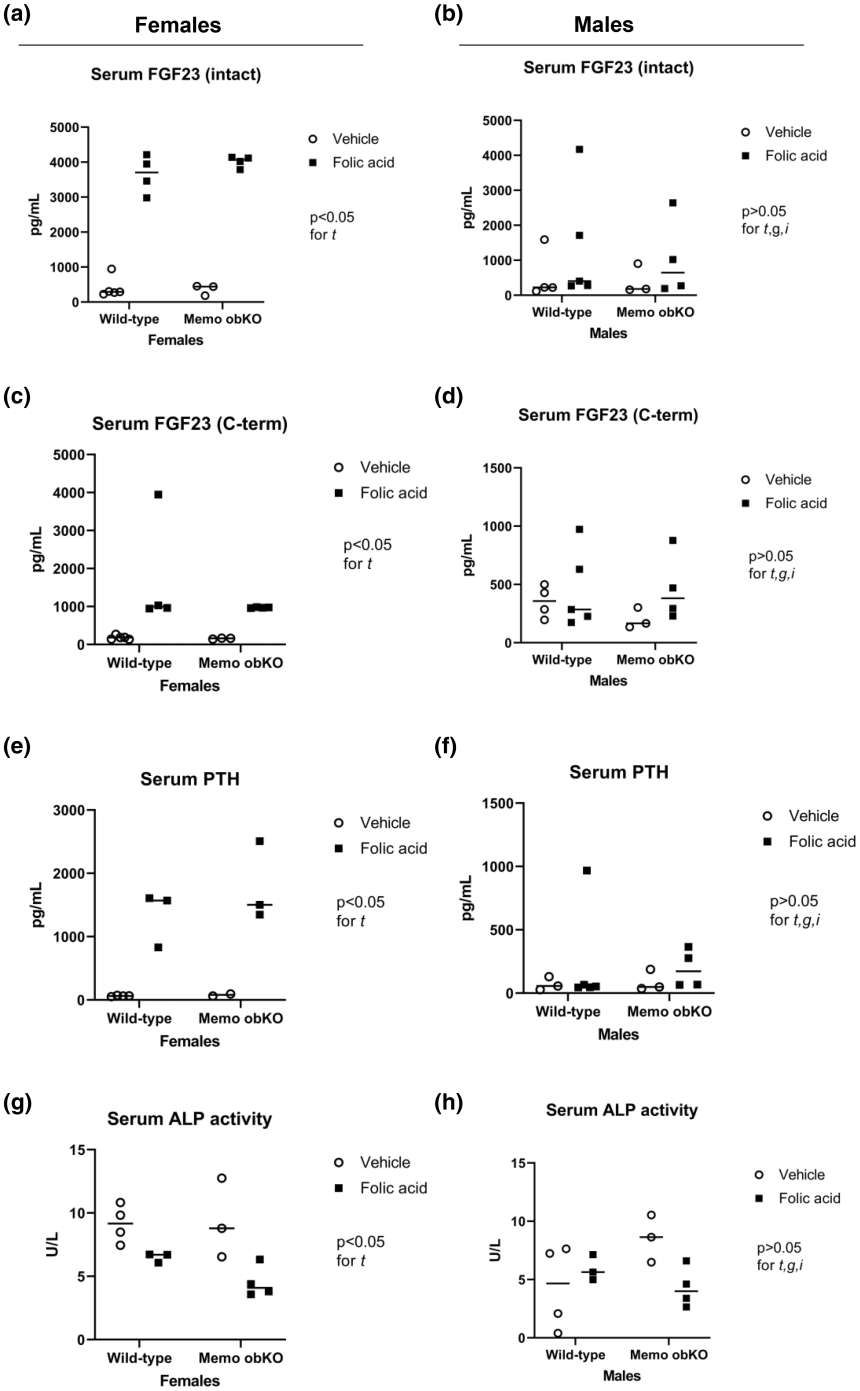
Circulating mineralotropic hormones in AKI influenced by sex, but not by genotype. Circulating intact FGF23 (a and b), C‐terminal FGF23 (c and d), PTH (e and f), and serum alkaline phosphatase activity (g and h) showed patterns of significant treatment effects in females but not in males. PTH, parathyroid hormone. ALP, alkaline phosphatase activity. Statistical analyses by Two‐ANOVA within each sex. t, treatment effect (folic acid versus vehicle); g, genotype effect; i, interaction between treatment and genotype. N of animals is indicated by the number of scatters. Age of mice was 9 weeks. Data of both sexes pooled are shown in Figure S5.

Experimental AKI caused a 5‐fold increase in *Fgf23* expression in calvaria of control genotype (both sexes combined: *p* < 0.05) and a trend to a 2.5‐fold increase in Memo1 obKO mice (Figure 5a,b, Figure S4a). By contrast *Fgf23* gene expression in bone marrow‐free femur (Figure 5b,c, Figure S4b) or in bone marrow (Figure 5e,f, Figure S4c) did not show any significant changes between the different treatment and genotype groups. ELISA analyses of serum C‐terminal, intact FGF23, and PTH displayed significant differences between sexes. Circulating intact FGF23, C‐terminal FGF23, and PTH were increased after folic acid injection in both Memo1 obKO and control mice; however, their increase was significant in females only and considerably higher than in males (Figure 6a–f, Figure S5a–c). Analyses of serum ALP activity revealed a negative folic acid treatment effect in females, with lower values in mice with AKI (Figure 6g,h, Figure S5d).

To summarize, the absence of Memo1 in osteoblasts did not affect gross anatomy and bone structure. Moreover, in AKI, Memo1 does not significantly alter the development of the disease, but a heterogeneity of findings was present in sex‐disaggregated analyses that was in line with the renal findings of kidney injury biomarker Kim‐1.

## 4 DISCUSSION

Here, we set out to describe the role of FGFR modulator Memo1 in the osteoblast and in bone‐derived FGF23 expression under experimental AKI, based on reports that FGFR‐Klotho signaling is required for FGF23 expression during chronic kidney disease in mice (Kaludjerovic et al., 2017). We have generated a novel osteoblast‐specific Memo1 KO mouse model by crossing mice carrying loxP sites flanking *Memo1* exon 2 and a *Cre* recombinase under control of the α1(I)‐collagen promoter (Dacquin et al., 2002; Haenzi et al., 2014). A diminished of *Memo1* gene expression and Memo1 protein from total femur was successfully verified, while other organs remained unaffected and their *Memo1* expression remained comparable to control mice. Memo1 obKO were viable without changes in gross anatomy, except for a transient post‐weaning weight difference in males. In the present model, traits including kyphosis, failure to thrive and abnormalities in mineral homeostasis or skeletal structure were not observed, in contrast to post‐weaning *Memo1* deletion in the whole body that we previously reported (Haenzi et al., 2014; Moor, Ramakrishnan, et al., 2018). Similarly, we have previously reported on a kidney‐specific model of *Memo1* deletion that did not reproduce renal failure that was seen upon post‐weaning whole‐body deletion of *Memo1* (Moor, Haenzi et al., 2018; Moor, Ramakrishnan, et al., 2018).

We next used a well‐studied experimental model of AKI (Yan, 2021) to determine the contribution of osteoblast Memo1 to *Fgf23* expression. This caused renal tubular necrosis, expression of a renal injury marker, and a decrease in *Klotho* expression similar as previously reported by Egli‐spichtig et al. (2018). However, we found no differences between control and Memo obKO genotypes regarding the consequences of AKI including *Fgf23* expression. This implies that during the inflammatory changes driven by folic acid overload, autocrine or paracrine FGF23 signaling via a canonical FGFR‐Klotho pathway may play only a minor role, in contrast to more chronic models of kidney failure driven by other pathophysiology (Kaludjerovic et al., 2017). In contrast, both decreasing renal clearance and an increase of cytokines such as TNF and TGFβ, might be the link between AKI and the excessive *Fgf23* expression in different lymphoid and parenchymatous organs contributing high circulating FGF23 levels (Egli‐spichtig et al., 2018).

Next, in serendipitous response to a recent call for further study (Yan, 2021), we here report signs of sex differences in this experimental model of AKI, with females expressing much higher renal injury markers in comparison to males, and corresponding AKI‐dependent effects on PTH and FGF23 were visible in females only but less so in males. Findings of sex differences are most well known in other experimental models of AKI, such as ischemia–reperfusion injury by transient renal artery clamping where female mice required longer ischemia time compared to males in order to establish the same severity of AKI, and even then, the systemic consequences were more dire in male than in female animals (Dixon et al., 2022; Soranno et al., 2022). In the same line, in a model of tunicamycin‐driven endoplasmic reticulum stress, kidneys of male mice were much more susceptible to injury than of females (Hodeify et al., 2013). Importantly, in humans AKI more frequently occurs in men than in women even after correcting for sex differences in some underlying risk factors (Loutradis et al., 2021). Similarly, with a potential exception of aminoglycoside nephrotoxicity (Moore et al., 1984), the population‐wide incidence of dialysis initiation due to AKI was higher in men than in women in a systematic review (Neugarten et al., 2018).

The present study contains some limitations. First, we assessed bone structure but did not assess the morphology of bone by histomorphometry because no structural phenotype was present. Second, samples were underpowered to conclusively exclude potential differences in analytes of larger variability such as PTH, kalemia, or Conn.D. Next, we could not measure renal retention parameters or obtain renal histology in an adequately powered sample in order to conclusively establish the sex difference of AKI severity, which was not the original aim of the project. However, the measured renal biomarker Kim‐1 is a surrogate parameter of AKI severity (Bonventre, 2009), and the present data are a hint toward sex differences in this AKI model which should be followed up in further studies. Further, the inflammatory folic acid model used to drive AKI caused an increase in circulating C‐terminal and intact FGF23, but *Fgf23* expression was only increased in calvaria but not long bones. Similarly, others have previously reported a much higher degree of *Fgf23* expression increase in calvaria than in long bones (Durlacher‐Betzer et al., 2018; Egli‐spichtig et al., 2018; Hassan et al., 2016). This implies that distinct site‐dependent differences in the regulation of *Fgf23* expression by the bone may exist that should be addressed in targeted studies, similar as the anatomical differences in bone energy metabolism inferred from site‐specific differences in glucose uptake (Suchacki et al., 2021). Finally, the lack of a phenotype in the bone of Memo obKO may be attributable to the mode of action chosen for Cre expression. For instance, a postnatally inducible Cre expression or a mesenchymal progenitor cell Cre expression may have revealed a structural phenotype that was not apparent in the present model due to some compensation process, as Memo1 has a demonstrated role in ossification (Van Otterloo et al., 2016).

Collectively, the present study reveals no evidence for a role of Memo1 in mature osteoblasts contributing to bone structure or the expression of FGF23 in acute inflammatory kidney disease. However, the present data points toward potential sex differences in experimental folic acid‐driven AKI, including gene expression changes and circulating FGF23 and PTH, that warrants in‐depth study.

## AUTHOR CONTRIBUTIONS

Matthias B. Moor conceived the study. Katalin Bartos and Matthias B. Moor participated in experimental design. Katalin Bartos performed experiments. Katalin Bartos and Matthias B. Moor participated in data analysis and interpretation. Matthias B. Moor acquired funding for the research. Matthias B. Moor supervised the research. Katalin Bartos and Matthias B. Moor wrote the manuscript. All authors critically read and commented on the manuscript and agreed to manuscript submission.

## CONFLICT OF INTEREST STATEMENT

All authors declare that they have no conflict of interest to report.

## Supporting information


**Figure S1.** Mice showed normal post‐weaning body weight for Memo obKO and control genotype for both sexes. Error bar indicates standard deviation. *N* = 4 per genotype and sex
**Figure S2**. Sex‐aggregated data of Figure 3a,b. t, treatment effect in two‐way ANOVA.
**Figure S3**. Sex‐aggregated data of Figure 4a,b (a), 4c,d (b) and 4e,f (c). t, treatment effect; g, genotype effect; i, interaction in two‐way ANOVA.
**Figure S4**. Sex‐aggregated data of Figure 5a,b (a), 5c,d (b) and 5e,f (c). t, treatment effect; g, genotype effect; i, interaction in two‐way ANOVA.
**Figure S5**. Sex‐aggregated data of Figure 6a,b (a), 6c,d (b), 6e,f (c) and 6g,h (d). t, treatment effect; i, interaction in two‐way ANOVA.
