# Supplementary material for: FGFR regulator Memo1 is dispensable for FGF23 expression by osteoblasts during folic acid‐driven kidney injury
Source: Physiol Rep. 2023 Mar 26;11(6):e15650. doi: 10.14814/phy2.15650 (PMC10040316; doi:10.14814/phy2.15650)
Supplement: Supplementary file 1 — Figure S1. Mice showed normal post‐weaning body weight for Memo obKO and control genotype for both sexes. Error bar indicates standard deviation. N = 4 per genotype and sex Figure S2. Sex‐aggregated data of Figure 3a,b. t, treatment effect in two‐way ANOVA. Figure S3. Sex‐aggregated data of Figure 4a,b (a), 4c,d (b) and 4e,f (c). t, treatment effect; g, genotype effect; i, interaction in two‐way ANOVA. Figure S4. Sex‐aggregated data of Figure 5a,b (a), 5c,d (b) and 5e,f (c). t, treatment effect; g, genotype effect; i, interaction in two‐way ANOVA. Figure S5. Sex‐aggregated data of Figure 6a,b (a), 6c,d (b), 6e,f (c) and 6g,h (d). t, treatment effect; i, interaction in two‐way ANOVA. [file PHY2-11-e15650-s001.pdf]

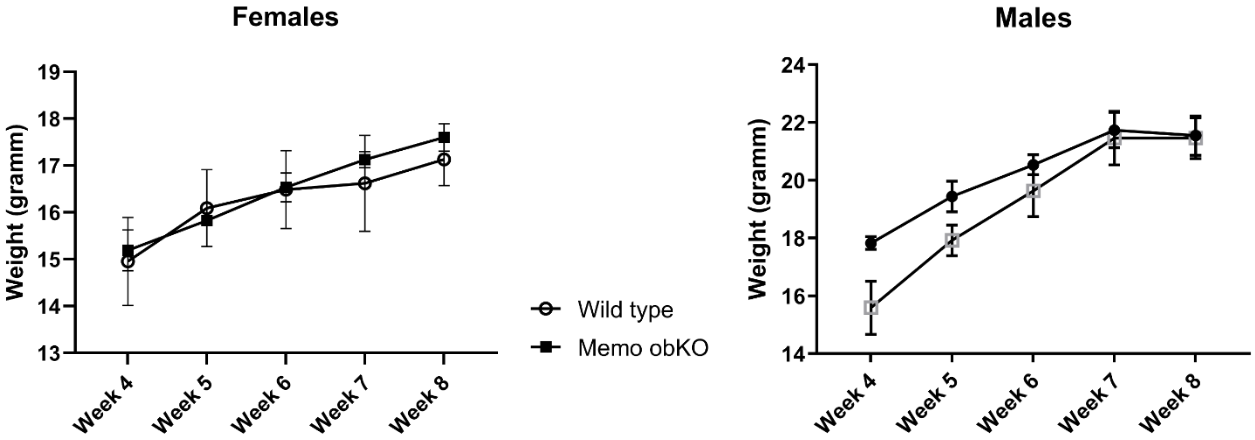


**Supplemental figure 1.** Mice showed normal post-weaning body weight for Memo obKO and control genotype for both sexes. Error bar indicates standard deviation. N=4 per genotype and sex.

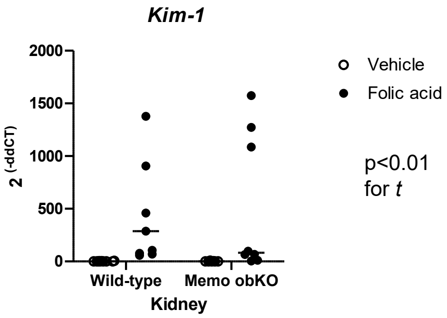


**Supplemental figure 2.** Sex-aggregated data of Figure 3A and B. t, treatment effect in Two-way ANOVA.

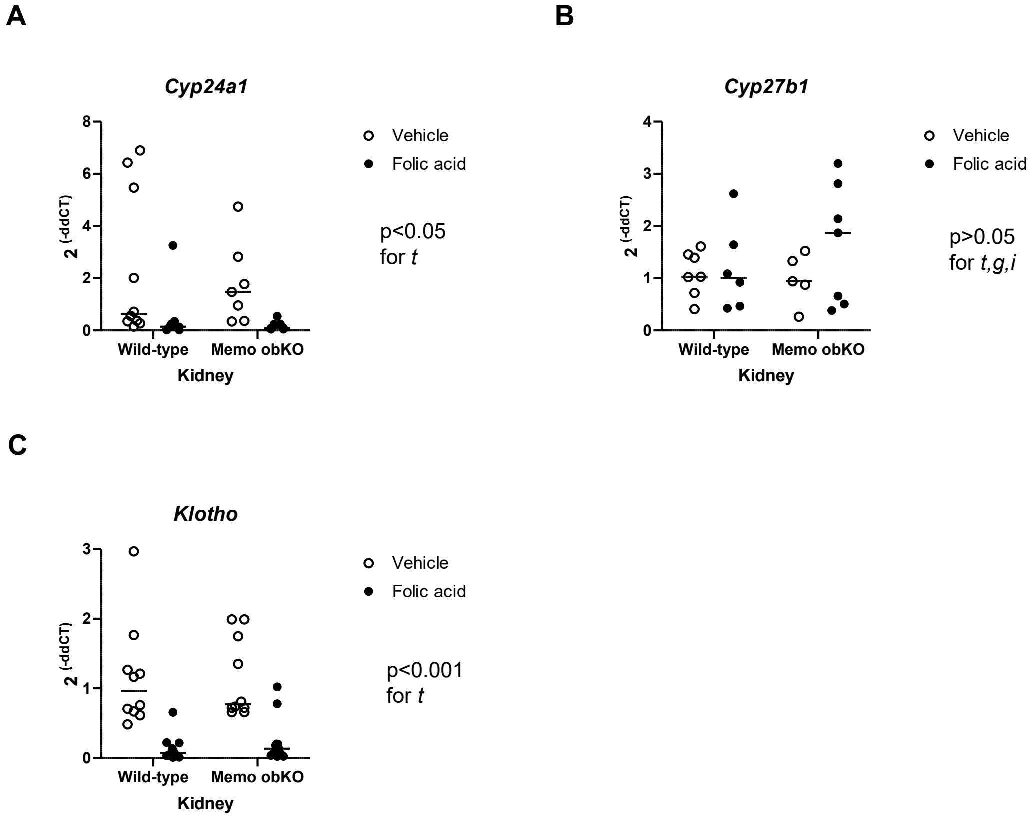


**Supplemental figure 3.** Sex-aggregated data of Figure 4A-B (A), 4C-D (B) and 4E-F (C). t, treatment effect; g, genotype effect; i, interaction in Two-way ANOVA.

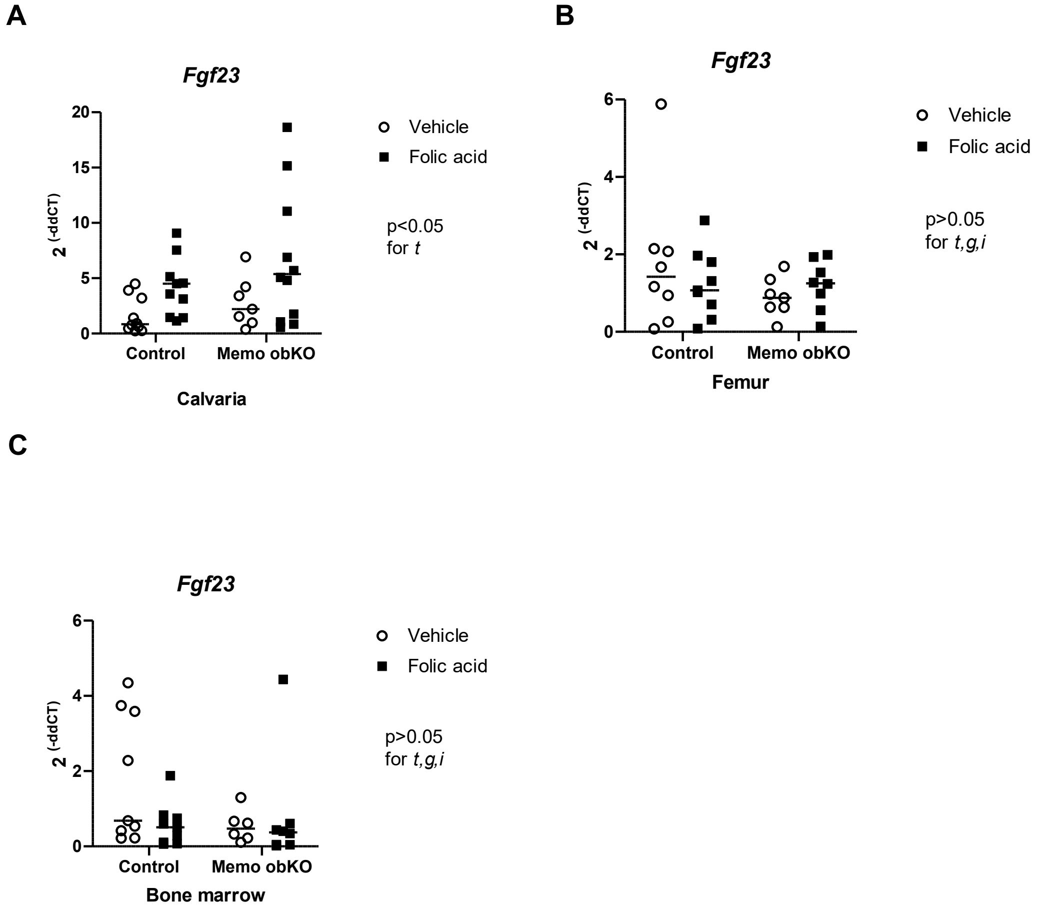


**Supplemental figure 4.** Sex-aggregated data of Figure 5A-B (A), 5C-D (B) and 5E-F (C). t, treatment effect; g, genotype effect; i, interaction in Two-way ANOVA.

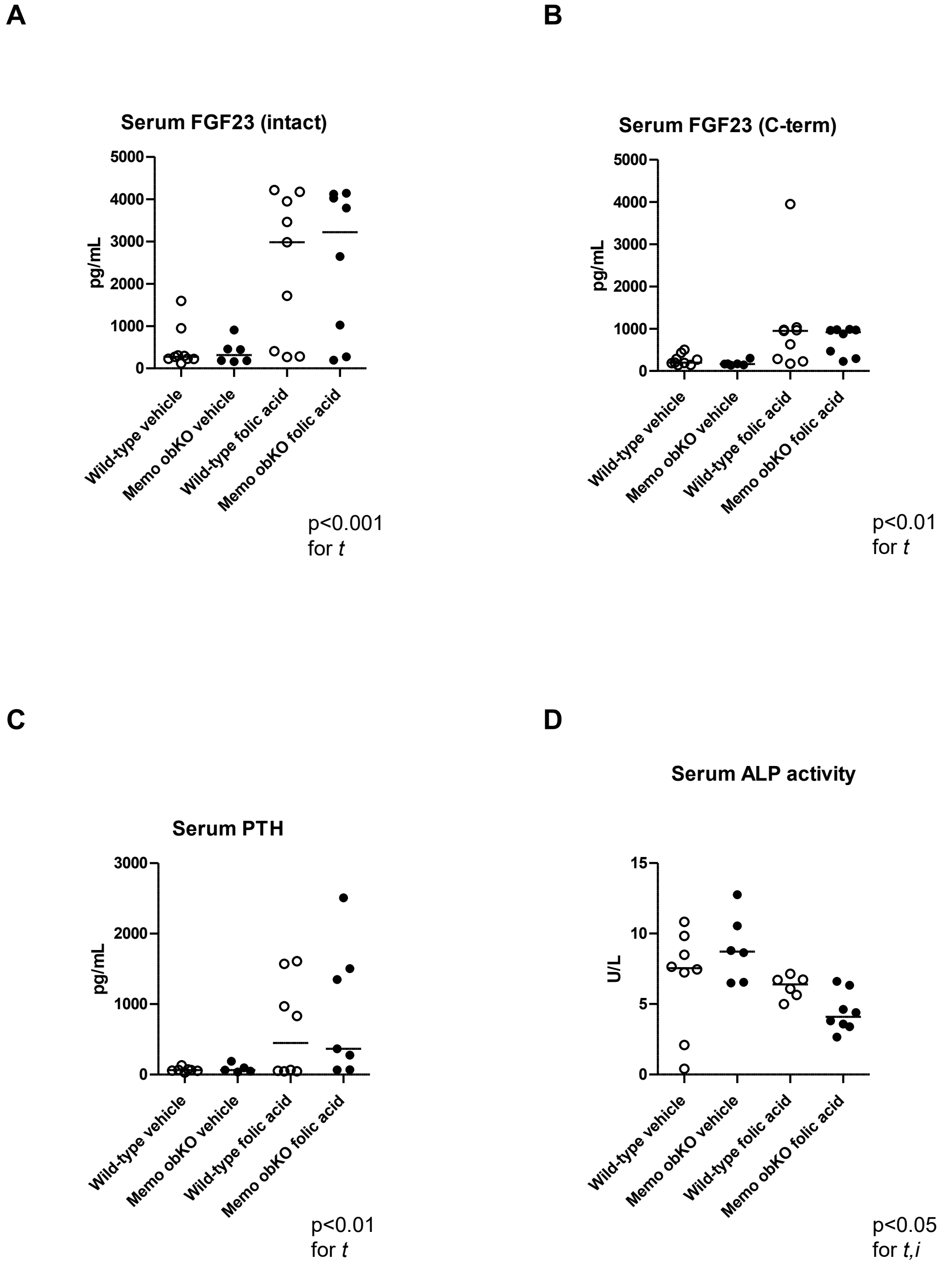


**Supplemental figure 5.** Sex-aggregated data of Figure 6A-B (A), 6C-D (B), 6E-F (C) and 6G-H (D). t, treatment effect; i, interaction in Two-way ANOVA.
